# Proximate Composition, Nutritional Attributes and Mineral Composition of *Peperomia pellucida* L. (Ketumpangan Air) Grown in Malaysia

**DOI:** 10.3390/molecules170911139

**Published:** 2012-09-17

**Authors:** Der-Jiun Ooi, Shahid Iqbal, Maznah Ismail

**Affiliations:** 1 Laboratory of Molecular Biomedicine, Institute of Bioscience, Universiti Putra Malaysia, UPM Serdang 43400, Selangor, Malaysia; Email: ooiderjiun@gmail.com (D.-J.O.); ranashahid313@gmail.com (S.I.); 2 Department of Chemistry, University of Sargodha, Sargodha 40100, Pakistan; 3 Department of Nutrition and Dietetic, Faculty of Medicine and Health Sciences, Universiti Putra Malaysia, UPM Serdang 43400, Selangor, Malaysia

**Keywords:** *Peperomia pellucida*, proximate composition, mineral profile

## Abstract

This study presents the proximate and mineral composition of *Peperomia pellucida* L., an underexploited weed plant in Malaysia. Proximate analysis was performed using standard AOAC methods and mineral contents were determined using atomic absorption spectrometry. The results indicated *Peperomia pellucida* to be rich in crude protein, carbohydrate and total ash contents. The high amount of total ash (31.22%) suggests a high-value mineral composition comprising potassium, calcium and iron as the main elements. The present study inferred that *Peperomia pellucida* would serve as a good source of protein and energy as well as micronutrients in the form of a leafy vegetable for human consumption.

## 1. Introduction

*Peperomia pellucida* L. (Piperaceae), a common annual weed native to tropical North and South America, is now pantropic in distribution and is abundantly available in Malaysia. Locally, it is variously known as sirih cina, ketumpangan air, tumpang angin or căo hú jīao. In other parts of the world, it is known as pak-krasang (Thailand), càng cua (Vietnam), pansit-pansitan or ulasimang bato (Philippines), silverbush, man-to-man, pepper elder or rat ear (North America), kaca-kaca or surukan (Indonesia), usuba sunakosho (Japan), alumbre or erva-de-vidro (Spain), mashitandu chedi, pononoa, toyakandha or varshabhoo (India), pépéromie or herbe à couleuvre (France) and luchi pata (Bangladesh). The plant flourishes in moist as well as in shady areas and can grow up to height of 15 to 45 cm. *P. pellucida* is characterized by its smooth, fleshy and heart-shaped leaves, succulent stems, shallow roots and minute flowers, which eventually grow into numerous tiny seeds attaching on the cord-like spikes [1]. 

The plant has been widely used for different medicinal purposes and has been used culturally for a variety of ailments including conjunctivitis, convulsions, fatigue, fever, headache, gout, rheumatic pains, skin diseases, to lower blood cholesterol level and breast cancer [1]. Its properties have been further affirmed by a number of research reports. Aerial parts of *P. pellucida* demonstrated dose-dependent analgesic and anti-inflammatory actions by interfering with prostaglandin synthesis [2,3,4]. Khan *et al*. [5,6] reported a dose-dependent depressant effect and antipyretic effect of *P. pellucida* leaf comparable to standard aspirin, while Mutee *et al*. [4] presented the potential use of the plant as an antioxidative agent. Nwokocha *et al.* [7] suggested a dose-dependent hypotensive effect of *P. pellucida* via enhancement of endothelial nitric oxide (NO)-dependent vasorelaxation. *P. pellucida* leaf also exhibited broad spectrum bactericidal [8] and fungicidal activity against *Helminthosporium oryzae* [9] and *Trichophyton mentagrophytes* [10].

Previous phytochemical analyses on this plant revealed the presence of flavonoids, apiols, phytosterols, substituted styrenes, secolignans, tetrahydrofuran lignans, arylpropanoids, sesamin, isoswertisin, xanthone glycoside and peperomins A, B, C and E in it. Arylpropanoids, peperomins and patuloside A (which is a type of xanthone glycoside) exhibited antifungal, anticancer and antibacterial activities, respectively [10,11,12]. 

It is an edible plant and is frequently used as a leafy vegetable or condiment in many parts of the tropics. Egwuche *et al.* [13] reported the proximate and mineral composition of *P. pellucida* leaf. Although some preliminary information has been presented in an earlier report, but detailed investigations are still required as *P. pellucida* is being consumed on a major scale and is traditionally prescribed as folk medicine in the whole plant form. No report on the nutritional and mineral profile of *P. pellucida*, indigenous to Malaysia, has been presented so far. Nutritional composition and phytochemical properties are reported to vary with agroclimatic conditions, humidity and species of the plant [14]. This study was, therefore, carried out to determine the proximate composition and mineral contents in *P. pellucida* whole plant collected from Malaysia.

## 2. Results and Discussion

The findings for proximate composition of *P. pellucida* whole plant are presented in Table 1. The moisture content of fresh whole plant *P. pellucida* was found to be 93.14%, contributed by the bulk tissue weight of fleshy and succulent stem. After oven drying, the *P. pellucida* whole plant still contained a moisture level with a mean value of 8.33%. The high water content in *P. pellucida* makes it ideal for vegetable juicing as a supplement to whole vegetables. Nevertheless, in terms of natural product stability, high moisture content tends to promote microbial contamination and chemical degradation [15], as it provides a medium for many reactions to occur. 

**Table 1:** Proximate composition of *Peperomia pellucida*.

Analysis	Mean ± SD
Proximate analysis (g/100 g DW)	
Moisture	8.33 ± 0.33
Protein	10.63 ± 0.07
Lipid	3.24 ± 0.28
Carbohydrates	46.58 ± 2.74
Total ash	31.22 ± 2.06
Caloric value (kcal/kJ)	258/1080

Each value represents the mean ± SD of three determinations on dry weight (DW) basis.

The protein and lipid contents of the whole plant were 10.63 and 3.24%, respectively. Protein content of *P. pellucida* is comparable to that of other leafy vegetables, where the protein content ranged from 1 to 7% of fresh weight or 8 to 30% of dry weight basis [16]. The major components of *P. pellucida* oil had been reported to be dillapiole and *trans*-caryophyllene [17,18] where *trans*-caryophyllene had previously exhibited anti-inflammatory action in mice [19]. The caloric value of *P. pellucida* was found to be 258 kcal/100 g DW; indicating it could be an important source of energy. The carbohydrate level (46.58%) was the highest calorie contributor, as the total lipid and protein contents did not considerably affect the determination of energy produced.

The ash content is generally recognized as a measure of quality for the assessment of the functional properties of foods [20]. *P. pellucida* contained high levels of total ash, up to 31.22%, in the dried plant. This, in turn, indicated a high content of minerals such as potassium (6,977 mg/100 g DW), followed by calcium (483 mg/100 g DW) and iron (119.3 mg/100 g DW). The mineral profile of *P. pellucida* whole plant is shown in Table 2. The high content of potassium compared to sodium led to a very low Na/K ratio, which is favorable from nutritional point of view, as diets with low Na/K ratio are associated with lower incidence of hypertension [21]. This may explain the nitric oxide (NO)-dependent hypotensive effect reported by Nwokocha *et al*. [7] and the rationale behind the traditional use of the plant in managing hypertension [22]. Calcium was found to be the second most abundant mineral element present in this plant. Therefore *P. pellucida* can be considered an appropriate dietary source of calcium to maintain the biological role of nerve transmission, muscle contraction, glandular secretion as well as mediating vascular contraction and vasodilation [23].

Iron deficiency is a common nutritional problem affecting many people worldwide. Primarily, it occurs as a result of chronic bleeding, infections, inadequate intake of bioavailable iron, deficiencies of folic acid, vitamin A or vitamin B_12_, pregnancy, increased requirements throughout growing periods and menstrual losses in women during reproductive age. The high amount of iron found in *P. pellucida* indicated that the plant could be a good source of dietary iron to overcome nutritional deficiency of iron, if supplemented in the diet.

**Table 2:** Mineral composition of *Peperomia pellucida*.

Analysis	Mean ± SD
Minerals (mg/100 g DW)	
Potassium	6977 ± 4.24
Calcium	483 ± 97.02
Iron	119.3 ± 20.33
Sodium	53.92 ± 0.37
Zinc	12.59 ± 0.25
Copper	3.10 ± 0.33

Values are means ± SD calculated as milligram per 100 gram dry weight (DW) for *Peperomia pellucida* analyzed individually in triplicate.

Copper and zinc are the essential trace elements that are needed only in minute amounts by the human body for important biochemical functions. The levels of copper and zinc are closely interrelated. Zinc stimulates the synthesis of metallothionein. Metallothionein has a high affinity for copper and it hinders copper systemic absorption within the intestinal cells [24]. Excessive ratio of zinc to copper (>16) from dietary sources causes imbalance in their bioavailability and has been linked to increased risk of cardiovascular system disorders [25]. The recommended zinc/copper ratio in human tissues is four to six [26,27]. *P. pellucida*, with its zinc/copper ratio of four, represents a potential food source to counter copper-zinc imbalances.

## 3. Experimental

### 3.1. Procurement and Preparation of Plant Materials

*Peperomia pellucida* (L.) plant was harvested as one batch from Guar Chempedak, Kedah, Malaysia. The collected plant sample was washed under running tap water, rinsed with distilled water and oven-dried at 40 °C for 48 h using a material test chamber M720 (Binder GmbH, Tuttlingen, Germany). Obtained dry matter was ground to a fine powder, sifted in a sieve (35 mm) and stored at −20 °C in air-tight containers till further analyses.

### 3.2. Proximate Analyses

#### 3.2.1. Moisture

Moisture content was measured using air-oven following official methods of Association of Official Analytical Chemists [28]. A material test chamber M720 (Binder GmbH, Tuttlingen, Germany) was used to dry the samples till constant weight. The percentage of moisture content was calculated as:


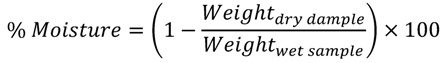



#### 3.2.2. Lipid

Determination of lipid content was performed following Soxtec method previously described by Noureddini and Byun [29], using a Soxtec^TM^ 2050 automated analyzer (FOSS Analytical, Hillerød, Denmark). Petroleum ether was used for the extraction, whereas percentage of lipid was obtained following equation below: 


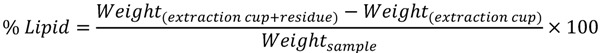


#### 3.2.3. Protein

The total nitrogen amount in the sample was determined according to method described by Ng *et al*. [30] using Kjeltec^TM^ 2200 Auto Distillation Unit (FOSS Tecator, Höganäs, Sweden). A nitrogen-to-protein conversion factor of 4.4 was used for the determination of protein present in the samples.

#### 3.2.4. Ash Content and Ash Solution

A dry ashing method was used to determine the ash content [28]. The samples were incinerated in a furnace (Furnace 62700, Barnstead/Thermolyne, Dubuque, IA, USA) at 550 °C. The remaining inorganic material was cooled, weighed and further used for the determination of mineral contents. An ash solution was prepared by dissolving the ash in 100 mL of 1 M HCl.

#### 3.2.5. Carbohydrate and Caloric Value

The total carbohydrate content (%) in the samples was calculated by difference method. The caloric value was calculated by sum of the percentages of proteins and carbohydrates multiplied by a factor of 4 (kcal/g) and total lipids multiplied by a factor of 9 (kcal/g).

### 3.3. Mineral Analyses

A NOVA 400 atomic absorption spectrometer (Analytik Jena AG, Jena, Germany) with an air/acetylene flame and respective hollow-cathode lamps was used for absorbance measurements. Wavelengths, slits and lamp current used for the determination of six elements were 213.9 nm, 0.5 nm, 4.0 mA (zinc); 422.7 nm, 1.2 nm, 4.0 mA (calcium); 324.8 nm, 1.2 nm, 3.0 mA (copper); 589.0 nm, 0.8 nm, 3.0 mA (sodium); 248.3 m, 0.2 nm, 6.0 mA (iron) and 766.5 nm, 0.8 nm, 4.0 mA (potassium), respectively. The results for mineral contents were expressed as mg/100 g DW.

### 3.4. Statistical Analysis

Statistical analyses were conducted using GraphPad Prism (San Diego, CA, USA) version 5.02 for Windows. All the determinations were carried out in triplicate and data were expressed as mean ± standard deviation.

## 4. Conclusions

In conclusion, the potential of *P. pellucida* as an important source of nutritional and mineral compounds in the tropics is highlighted in the present study. In line with increasing global demand for food, *P. pellucida* finds use as potential source of functional foods.

*Sample* *Availability*: Samples are available from the authors. 
